# Mechanism of Common-mode Noise Generation in Multi-conductor Transmission Lines

**DOI:** 10.1038/s41598-019-51259-w

**Published:** 2019-10-21

**Authors:** Souma Jinno, Shuji Kitora, Hiroshi Toki, Masayuki Abe

**Affiliations:** Graduate School of Engineering Science, Osaka University, Toyonaka, Osaka 560-8531 Japan

**Keywords:** Electrical and electronic engineering, Physics

## Abstract

The mechanism of common-mode noise generation in multi-conductor transmission lines is presented. Telegraphic equations, wave equations, and reflection coefficients in the normal and common modes are derived, which provide the mechanism of common-mode noise generation. In addition to coupling among transmission lines, the origin of the common-mode noise generation is elucidated by deriving the reflection coefficients in the normal and common modes.

## Introduction

In the fabrication of electric and electronic circuits, noise analysis is an important process to improve the performance of original designs. Electromagnetic (EM) noise among the transmission lines in the propagation of electric signals is a troublesome problem. Although multi-conductor transmission line (MTL) theory should enable the interference of signals among distributed lines to be described^1–10^, there remain difficulties in obtaining an intuitive interpretation of noise and in reducing the noise significantly.

From a physical point of view, electromagnetic noise originates from interactions between a circuit and its environment, such as the ground and the earth. However, the environment in an MTL system is usually represented theoretically as an ideal reference frame, which does not have any physical function. It is important to treat conductors in the environment explicitly for the quantitative understanding of electromagnetic noise. If we approximate the conductors in the environment as a transmission line, we are able to formulate a telegraph equation representing potentials and currents in MTLs from the Maxwell equations^11^. In a previous study, the concepts of the normal mode (NM) and the common mode (CM) were introduced in order to obtain an intuitive understanding of EM noise. The NM is usually treated as a circuit signal, and the CM is generated by interaction with the environment and causes various noises. Originally, the concepts of the NM and CM voltages were discussed by Maxwell in his book^12^. He pointed out that the observable quantity is the normal mode, which is the differential mode, and did not pay much attention to the common mode, even saying that the common mode had “no physical meaning”. However, these two modes interact with each other, and electric signals are interchanged from one to the other^13,14^. This interaction would induce CM noise and even generate unwanted external radiation^15–19^.

One of the successful cases in actual applications to reduce noise is a power supply of accelerators for particle physics^20,21^. The power supplies have a symmetrical configuration of three transmission lines together with lumped circuits, which suppresses the interference of the normal and common modes in the power lines^22^. The symmetric three-line circuit was verified to be the best configuration for the reduction of noise, where the normal mode decouples from the common mode^11^. We formulated a three-line circuit for numerical calculations and showed the relation between the CM noise^23^ and can say that electrically and geometrically symmetric circuit structure does not generate CM noise and does not couple with the normal mode^24^.

Previously, we showed numerically why the symmetric three-line circuit is the best configuration for noise reduction. However, it is desirable to understand why this configuration is the best and to know what is important to the actual choice of electric components. To this end, we would like to formulate the reflection coefficients of the three-line circuit for any electric components to be attached to the three-line circuit. In this way, we are able to quantify the amount of common mode noise in three-line circuits.

In the present paper, we introduce an analytical approach that supports further understanding of the electromagnetic noise induced in three-conductor transmission-line circuits. In Section 2, we introduce a formulation in the NM and CM framework for all of the quantities describing the circuit. We apply the formula to three-line systems and define vectors and matrices in the NM and CM quantities, which provide an intuitive understanding of the CM noise generation at the MTL boundaries. Equations and parameters of the signal propagation are expressed in the NM and CM framework. We found that coupling between the NM and CM modes occurs not only along the MTL, but also at the edges of the MLT, and the coupling at the edges of the MLT also depends on the lumped-parameter circuit configuration. In Section 3, we discuss the configurations of both the distributed- and lumped-parameter circuits for the reduction of the CM noise and calculate explicitly the amount of coupling for several circuit configurations using the formula developed in the present paper. Section 4 summarizes the present study.

## Transmission-Line Theory in Terms of the Normal and Common Modes of a Three-Line Circuit

### Telegraphic and wave equations

**Figure 1:**
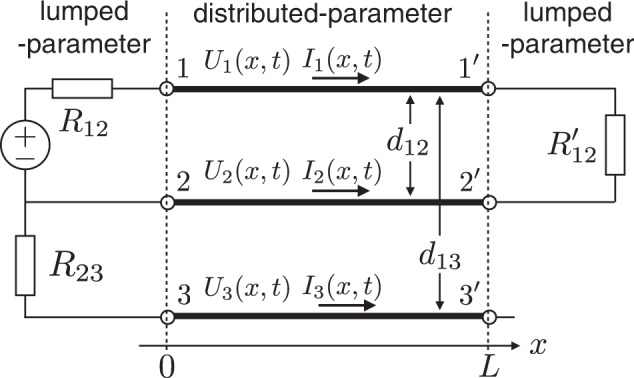
Three-line circuit consisting of three lines, 1 − 1′, 2 − 2′, and 3 − 3′, with resistances *R*_12_ and *R*_23_ and a power supply denoted by a circled ± on the source side and resistance *R*′_12_ on the load side.

In order to formulate reflection coefficients in the NM and CM, as in our previous studies^11^, we use a three-line circuit to which lumped-parameter circuits are connected, as shown in Fig. 1. In addition to a conventional two-line circuit configuration (distributed lines 1−1′ and 2−2′ with lumped parts), another conductor line, 3−3′, is connected on the source side as the ground. The NM is expressed as a relative relationship between two lines of the circuit and usually used in the circuit theory as “observable” difference mode:

1 $${V}_{{\rm{n}}}={U}_{1}-{U}_{2}$$

2 $${I}_{{\rm{n}}}=\frac{1}{2}({I}_{1}-{I}_{2})$$

Since the CM is generated by the interaction between the circuit and the surrounding environment, line 3−3′ should be explicitly considered as a conductor influencing the signal lines. In the same way as NM, the CM can be formulated by considering the difference mode between the circuit and the environment, where we express the circuit as the average potential and sum current of two lines^22^:

3 $${V}_{{\rm{c}}}=\frac{1}{2}({U}_{1}+{U}_{2})-{U}_{3}$$

4 $${I}_{{\rm{c}}}=\frac{1}{2}({I}_{1}+{I}_{2}-{I}_{3})$$

The NM-CM expression of the potentials and currents under the TEM approximation leads to telegraphic equations for the NM-CM quantities^11^.

5 $$\frac{\partial {V}_{{\rm{n}}}(x,t)}{\partial t}=-{P}_{{\rm{nn}}}\frac{\partial {I}_{n}(x,t)}{\partial x}-{P}_{{\rm{nc}}}\frac{\partial {I}_{{\rm{c}}}(x,t)}{\partial x},$$

6 $$\frac{\partial {V}_{{\rm{c}}}(x,t)}{\partial t}=-{P}_{{\rm{cn}}}\frac{\partial {I}_{n}(x,t)}{\partial x}-{P}_{{\rm{cc}}}\frac{\partial {I}_{{\rm{c}}}(x,t)}{\partial x},$$

7 $$\frac{\partial {V}_{{\rm{n}}}(x,t)}{\partial x}=-{L}_{{\rm{nn}}}\frac{\partial {I}_{n}(x,t)}{\partial t}-{L}_{{\rm{nc}}}\frac{\partial {I}_{{\rm{c}}}(x,t)}{\partial t},$$

8 $$\frac{\partial {V}_{{\rm{c}}}(x,t)}{\partial x}=-{L}_{{\rm{cn}}}\frac{\partial {I}_{n}(x,t)}{\partial t}-{L}_{{\rm{cc}}}\frac{\partial {I}_{{\rm{c}}}(x,t)}{\partial t}.$$

Here, the three lines are assumed to be lossless and are in a uniform medium, where both the permittivity and magnetic permeability are constant. Furthermore, we assume that there is no radiation from the circuit, *I*_1_ + *I*_2_ + *I*_3_ = 0^22^. Both *P* and *L* represent potential and inductance coefficients, respectively. We use *P* instead of capacitance, which is conventionally used in the one-dimensional telegraphic equation, because we need to consider potentials induced by charge for further discussion^25,26^. The subscripts denote the diagonal and coupling terms of the normal and common modes^11^.

The telegraphic equations indicate that the potentials and currents propagate in the conductor lines accompanying the NM and CM couplings, unless the decoupling condition *P*_cn_ = *P*_nc_ = *L*_cn_ = *L*_nc_ = 0 is satisfied. Physically, the decoupling conditions are that lines 1−1′ and 2−2′ have the same sizes and are positioned symmetrically with respect to line 3−3′^24^.

### Definitions of vectors and matrices in the NM-CM quantities

In order to understand the NM-CM coupling, we define the vectors and matrices in the NM-CM quantities.

9 $$\begin{array}{c}{\bf{V}}(x,t)=(\begin{array}{c}{V}_{{\rm{n}}}(x,t)\\ {V}_{{\rm{c}}}(x,t)\end{array}),\,{\bf{I}}(x,t)=(\begin{array}{c}{I}_{{\rm{n}}}(x,t)\\ {I}_{{\rm{c}}}(x,t)\end{array}),\\ {\bf{P}}=(\begin{array}{cc}{P}_{{\rm{n}}{\rm{n}}} & {P}_{{\rm{n}}{\rm{c}}}\\ {P}_{{\rm{c}}{\rm{n}}} & {P}_{{\rm{c}}{\rm{c}}}\end{array}),\,{\bf{L}}=(\begin{array}{cc}{L}_{{\rm{n}}{\rm{n}}} & {L}_{{\rm{n}}{\rm{c}}}\\ {L}_{{\rm{c}}{\rm{n}}} & {L}_{{\rm{c}}{\rm{c}}}\end{array}).\end{array}$$

Here, **V** and **I** are vectors of the NM and CM quantities, respectively. In addition, **P** and **L** are matrices of the potential and inductance coefficients, which were derived in a previous study^11^.

Definitions of the respective vectors and matrices provide wave equations in the NM-CM quantities.

10 $$\frac{{\partial }^{2}}{\partial {x}^{2}}{\bf{V}}(x,t)=\frac{1}{{c}^{2}}\frac{{\partial }^{2}}{\partial {t}^{2}}{\bf{V}}(x,t),$$

11 $$\frac{{\partial }^{2}}{\partial {x}^{2}}{\bf{I}}(x,t)=\frac{1}{{c}^{2}}\frac{\partial }{\partial {t}^{2}}{\bf{I}}(x,t\mathrm{).}$$

Here, *c* is the signal velocity in the MTL, and the coefficient matrices satisfy the relation **LP**^−1^ = **P**^−1^ **L** = 1/*c*^2^1, where **1** is an identity matrix^22^.

**Figure 2:**
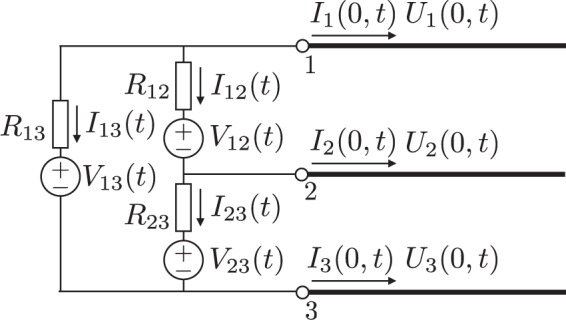
Lumped-parameter circuit consisting of resistances and voltage sources connected to a three-line MTL. By substituting *V*_13_ = *V*_23_ = 0 and *R*_13_ → ∞, the circuit becomes equal to the source (left) side of Fig. 1. The right-hand boundary circuit of Fig. 1 will be obtained by setting *V*_12_ = *V*_13_ = *V*_23_ = 0 and *R*_12_ → ∞ and *R*_13_ → ∞.

The reflection coefficients at the boundaries of the MTL depend both on the MTL configurations and on the elements of the lumped circuits connected to the MTL. Here, in order to derive the reflection coefficients in the NM-CM quantities, we study a case in which resistances and voltage sources are connected to the edges of the three-line circuit, as shown in Fig. 2. Here, we derive the NM and CM equations of the lumped circuit which is the boundary of MTL by using Kirchhoff’s equations and Ohm’s law. First, the currents flowing at the boundary (*I*_1_(0, *t*), *I*_2_(0, *t*) and *I*_3_(0, *t*)) are expressed by brunch currents in the lumped circuit:

12 $${I}_{1}(0,t)=-{I}_{13}(t)-{I}_{12}(t)$$

13 $${I}_{2}(0,t)={I}_{12}(t)-{I}_{23}(t)$$

14 $${I}_{3}(0,t)={I}_{23}(t)+{I}_{13}(t)$$

Next, the potentials at the boundary (*U*_1_(0, *t*), *U*_2_(0, *t*) and *U*_3_(0, *t*)) are also expressed by the lumped circuit quantities which are resistances, brunch currents and voltage sources:

15 $${U}_{1}(0,t)-{U}_{2}(0,t)={R}_{12}{I}_{12}(t)+{V}_{12}(t)$$

16 $${U}_{2}(0,t)-{U}_{3}(0,t)={R}_{23}{I}_{23}(t)+{V}_{23}(t)$$

17 $${U}_{1}(0,t)-{U}_{3}(0,t)={R}_{13}{I}_{13}(t)+{V}_{13}(t)$$

We can now rewrite these boundary conditions in terms of the NM and CM voltages and currents expressed in Eqs (1, 2, 3, 4). We write the results in a matrix form as:

18 $${\bf{V}}(x=\mathrm{0,}\,t)=-\,{\bf{RI}}\mathrm{(0,}\,t)+{\bf{E}}(t\mathrm{).}$$

Here, **R** and **E**(*t*) are a resistance matrix and a voltage source vector in the NM and CM quantities, respectively. Then, **R** is given as

19 $${\bf{R}}=(\begin{array}{cc}{R}_{{\rm{n}}{\rm{n}}} & {R}_{{\rm{n}}{\rm{c}}}\\ {R}_{{\rm{c}}{\rm{n}}} & {R}_{{\rm{c}}{\rm{c}}}\end{array}),$$

20 $${R}_{{\rm{nn}}}=\frac{{R}_{12}({R}_{23}+{R}_{13})}{{R}_{12}+{R}_{23}+{R}_{13}},$$

21 $${R}_{{\rm{cc}}}=\frac{1}{4}\frac{4{R}_{23}{R}_{13}+{R}_{12}{R}_{23}+{R}_{13}{R}_{12}}{{R}_{12}+{R}_{23}+{R}_{13}},$$

22 $${R}_{{\rm{nc}}}={R}_{{\rm{cn}}}=\frac{1}{2}\frac{{R}_{12}({R}_{13}-{R}_{23})}{{R}_{12}+{R}_{23}+{R}_{13}}.$$

Here, **E**(*t*) is expressed as

23 $${\bf{E}}={\bf{R}}{{\bf{I}}}_{S},$$

24 $${{\bf{I}}}_{S}=(\begin{array}{c}{V}_{12}/{R}_{12}-{V}_{23}/2{R}_{23}+{V}_{13}/2{R}_{13}\\ {V}_{23}/{R}_{23}+{V}_{13}/{R}_{13}\end{array}).$$

As for the lumped-parameter part at *x* = *L*, the relations of the lumped-parameter part connecting the nodes can be transformed into the NM and CM quantities in a similar manner.

25 $${\bf{V}}(x=L,t)={\bf{RI}}(L,t)+{\bf{E}}(t).$$

### Reflection coefficients

In a manner usually used in the recursive solution for the MTL^7^, we use boundary conditions that satisfy Eqs (10), (11), (18), and (25) at both edges (*x* = 0, *L*), and obtain the time-domain expression for **V**:

26 $$\begin{array}{rcl}{\bf{V}}\mathrm{(0,}\,t) & = & {{\bf{M}}}_{0}{{\bf{V}}}_{0}(t)\\  &  & +\mathrm{(1}+{{\boldsymbol{\Gamma }}}_{0})[{{\bf{M}}}_{L}{{\bf{V}}}_{L}(t-{T}_{D})+{{\boldsymbol{\Gamma }}}_{L}{{\boldsymbol{\Gamma }}}_{0}{{\bf{M}}}_{L}{{\bf{V}}}_{L}(t-3{T}_{D})\\  &  & \,\,\,\,\,\,+\cdots +{({{\boldsymbol{\Gamma }}}_{L}{{\boldsymbol{\Gamma }}}_{0})}^{N}{{\bf{M}}}_{L}{{\bf{V}}}_{L}(t-\mathrm{(2}N+\mathrm{1)}{T}_{D})]\\  &  & +\mathrm{(1}+{{\boldsymbol{\Gamma }}}_{0}){{\boldsymbol{\Gamma }}}_{L}[{{\bf{M}}}_{0}{{\bf{V}}}_{0}(t-2{T}_{D})+{{\boldsymbol{\Gamma }}}_{0}{{\boldsymbol{\Gamma }}}_{L}{{\bf{M}}}_{0}{{\bf{V}}}_{0}(t-4{T}_{D})\\  &  & \,\,\,\,\,\,+\cdots +{({{\boldsymbol{\Gamma }}}_{0}{{\boldsymbol{\Gamma }}}_{L})}^{N}{{\bf{M}}}_{S}{{\bf{V}}}_{0}(t-\mathrm{2(}N+\mathrm{1)}{T}_{D})]\end{array}$$

27 $$\begin{array}{rcl}{\bf{V}}(L,t) & = & {{\bf{M}}}_{L}{{\bf{V}}}_{L}(t)\\  &  & +\mathrm{(1}+{{\boldsymbol{\Gamma }}}_{L}){{\boldsymbol{\Gamma }}}_{0}[{{\bf{M}}}_{L}{{\bf{V}}}_{L}(t-2{T}_{D})+{{\boldsymbol{\Gamma }}}_{L}{{\boldsymbol{\Gamma }}}_{0}{{\bf{M}}}_{L}{{\bf{V}}}_{L}(t-4{T}_{D})\\  &  & \,\,\,\,\,\,+\cdots +{({{\boldsymbol{\Gamma }}}_{L}{{\boldsymbol{\Gamma }}}_{0})}^{N}{{\bf{M}}}_{L}{{\bf{V}}}_{L}(t-\mathrm{2(}N+\mathrm{1)}{T}_{D})]\\  &  & \,+\mathrm{(1}+{{\boldsymbol{\Gamma }}}_{L})[{{\bf{M}}}_{0}{{\bf{V}}}_{0}(t-{T}_{D})+{{\boldsymbol{\Gamma }}}_{0}{{\boldsymbol{\Gamma }}}_{L}{{\bf{M}}}_{0}{{\bf{V}}}_{0}(t-3{T}_{D})\\  &  & \,\,\,\,\,\,+\cdots +{({{\boldsymbol{\Gamma }}}_{0}{{\boldsymbol{\Gamma }}}_{L})}^{N}{{\bf{M}}}_{0}{{\bf{V}}}_{0}(t-\mathrm{(2}N+\mathrm{1)}{T}_{D})]\end{array}$$

Here, the subscripts for vectors and matrices 0 and *L* denote positions *x* = 0 and *x* = *L* of the MTL, respectively. In addition, *T*_*D*_ is the time for a signal to propagate from one end to the other. Although the amplitude of the reflection wave depends on characteristic impedances and resistances, we introduce matrices **M** and **Γ** in Eqs (26) and (27) in order to express the equations in the same form as the reflection of ordinary transmission line theory^7^.

28 $${\bf{M}}={\bf{Z}}{({\bf{R}}+{\bf{Z}})}^{-1}$$

29 $${\boldsymbol{\Gamma }}=({\bf{R}}-{\bf{Z}})({\bf{R}}+{\bf{Z}}{)}^{-1}$$

Here, **Z** is the impedance matrix in the NM-CM framework and is given as

30 $${\bf{Z}}=(\begin{array}{cc}{Z}_{{\rm{n}}{\rm{n}}} & {Z}_{{\rm{n}}{\rm{c}}}\\ {Z}_{{\rm{c}}{\rm{n}}} & {Z}_{{\rm{c}}{\rm{c}}}\end{array})={\bf{P}}/c={\bf{L}}c.$$

Note that the introduction of these matrices allows us to discuss wave reflection in the NM-CM framework in the standard way. Formulations of these matrices are similar to those defined in the standard MTL theory^7,9^: Here, **M** and **Γ** correspond to the voltage division coefficient and the reflection coefficient. Matrix elements of the voltage division **M** and the reflection **Γ** are given as follows:

31 $${\bf{M}}=(\begin{array}{cc}{M}_{{\rm{n}}{\rm{n}}} & {M}_{{\rm{n}}{\rm{c}}}\\ {M}_{{\rm{c}}{\rm{n}}} & {M}_{{\rm{c}}{\rm{c}}}\end{array}),$$

32 $${\boldsymbol{\Gamma }}=(\begin{array}{cc}{\Gamma }_{{\rm{n}}{\rm{n}}} & {\Gamma }_{{\rm{n}}{\rm{c}}}\\ {\Gamma }_{{\rm{c}}{\rm{n}}} & {\Gamma }_{{\rm{c}}{\rm{c}}}\end{array}).$$

These elements are derived from Eqs (28) and (29):

33 $${M}_{{\rm{nn}}}=\frac{1}{A}[{Z}_{{\rm{nn}}}({R}_{{\rm{cc}}}+{Z}_{{\rm{cc}}})-{Z}_{{\rm{nc}}}({R}_{{\rm{cn}}}+{Z}_{{\rm{cn}}})],$$

34 $${M}_{{\rm{n}}{\rm{c}}}=\frac{1}{A}[{Z}_{{\rm{n}}{\rm{c}}}{R}_{{\rm{n}}{\rm{n}}}-{Z}_{{\rm{n}}{\rm{n}}}{R}_{{\rm{n}}{\rm{c}}}],$$

35 $${M}_{{\rm{cn}}}=\frac{1}{A}[{Z}_{{\rm{cn}}}{R}_{{\rm{cc}}}-{Z}_{{\rm{cc}}}{R}_{{\rm{cn}}}],$$

36 $${M}_{{\rm{cc}}}=\frac{1}{A}[\,-\,{Z}_{{\rm{cn}}}({R}_{{\rm{nc}}}+{Z}_{{\rm{nc}}})+{Z}_{{\rm{cc}}}({R}_{{\rm{nn}}}+{Z}_{{\rm{nn}}})],$$

37 $${\Gamma }_{{\rm{nn}}}=\frac{1}{A}[({R}_{{\rm{nn}}}-{Z}_{{\rm{nn}}})({R}_{{\rm{cc}}}+{Z}_{{\rm{cc}}})-({R}_{{\rm{nc}}}-{Z}_{{\rm{nc}}})({R}_{{\rm{cn}}}+{Z}_{{\rm{cn}}})],$$

38 $${\Gamma }_{{\rm{nc}}}=\frac{2}{A}[{Z}_{{\rm{nn}}}{R}_{{\rm{nc}}}-{Z}_{{\rm{nc}}}{R}_{{\rm{nn}}}],$$

39 $${\Gamma }_{{\rm{cn}}}=\frac{2}{A}[{Z}_{{\rm{cc}}}{R}_{{\rm{cn}}}-{Z}_{{\rm{cn}}}{R}_{{\rm{cc}}}],$$

40 $${\Gamma }_{{\rm{c}}{\rm{c}}}=\frac{1}{A}[\,-\,({R}_{{\rm{c}}{\rm{n}}}-{Z}_{{\rm{c}}{\rm{n}}})({R}_{{\rm{n}}{\rm{c}}}+{Z}_{{\rm{n}}{\rm{c}}})+({R}_{{\rm{c}}{\rm{c}}}-{Z}_{{\rm{c}}{\rm{c}}})({R}_{{\rm{n}}{\rm{n}}}+{Z}_{{\rm{n}}{\rm{n}}})],$$

where

41 $$A=({R}_{{\rm{nn}}}+{Z}_{{\rm{nn}}})({R}_{{\rm{cc}}}+{Z}_{{\rm{cc}}})-({R}_{{\rm{nc}}}+{Z}_{{\rm{nc}}})({R}_{{\rm{cn}}}+{Z}_{{\rm{cn}}}).$$

Both **M** and **Γ** depend on **R**, which is determined by the lumped-parameter circuit connected to MTL at the boundary, and on **Z**, which is determined by the structure of the distributed-parameter circuit.

## Discussion on the Reduction of Electromagnetic Noise

The derivation of the above formulae provides insight for understanding CM noise generation in the MTL. There are two origins of CM noise generation. First, signal propagation induces CM noise in the MTL. The wave equations (Eqs (10) and (11)) show that the NM and CM current and voltage are not coupled with each other in appearance, even if the non-diagonal elements of **P** and **L** are not zero. Telegraph equations (Eqs (5) through (8)), however, exhibit NM-CM coupling. Note that coupling occurs between current and voltage in each mode. This means that, in CM noise generation, voltage-to-voltage or current-to-current coupling may not be so strong. The voltage-to-current (or current-to-voltage) conversion complicates understanding of the EM noise phenomena.

Next, CM noise generation occurs at the boundaries. The second term of Γ_nn_ indicates that NM-CM coupling also occurs at the boundaries. Common mode noise is always generated when the signal arrives at the boundaries, unless the coupling terms are zero. Although the condition of *R*_nc_ − *Z*_nc_ = 0 can decouple the two modes, it is actually almost impossible to adjust *R*_nc_ and *Z*_nc_ to be equal so as to satisfy these conditions.

**Figure 3:**
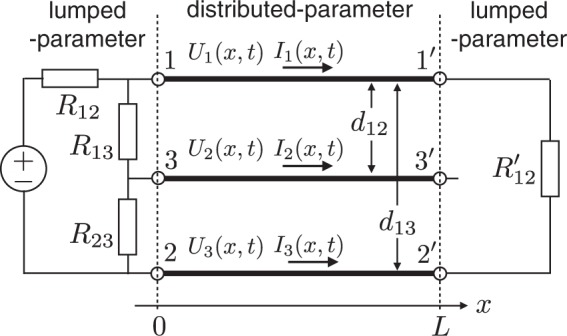
Three-line MTL circuit without NM and CM mode coupling.

One practical solution for NM-CM decoupling is to simultaneously satisfy *R*_nc_ = 0 and *Z*_nc_ = 0, which is satisfied by a symmetrical arrangement of the MTL and lumped elements, as shown in Fig. 3. Line 3−3′ is positioned symmetrically at the center between lines 1−1′ and 2−2′ (*d*_13_ = *d*_12_/2). The lumped-parameter parts should also be connected symmetrically to the MTL.

The matrix elements of **M** and **Γ** at both ends can be obtained by setting *V*_13_ = *V*_23_ = 0 and *R*_13_ = *R*_23_ on the source side, and *V*_12_ = *V*_13_ = *V*_23_ = 0, *R*_12_, *R*_13_ → ∞ and *R*′_12_ to be the characteristic impedance between lines 1−1′ and 2−2′ on the load side.

42 $${{\bf{M}}}_{0}=(\begin{array}{cc}{Z}_{{\rm{n}}{\rm{n}}}/({R}_{{\rm{n}}{\rm{n}}}+{Z}_{{\rm{n}}{\rm{n}}}) & 0\\ 0 & 0\end{array}),$$

43 $${{\boldsymbol{\Gamma }}}_{0}=(\begin{array}{cc}({R}_{{\rm{n}}{\rm{n}}}-{Z}_{{\rm{n}}{\rm{n}}})/({R}_{{\rm{n}}{\rm{n}}}+{Z}_{{\rm{n}}{\rm{n}}}) & 0\\ 0 & ({R}_{{\rm{c}}{\rm{c}}}-{Z}_{{\rm{c}}{\rm{c}}})/({R}_{{\rm{c}}{\rm{c}}}+{Z}_{{\rm{c}}{\rm{c}}})\end{array}),$$

44 $${{\bf{M}}}_{L}=(\begin{array}{cc}{Z}_{{\rm{n}}{\rm{n}}}/({R}_{{\rm{n}}{\rm{n}}}+{Z}_{{\rm{n}}{\rm{n}}}) & 0\\ 0 & 0\end{array}),$$

45 $${{\boldsymbol{\Gamma }}}_{L}=(\begin{array}{cc}0 & 0\\ 0 & 1\end{array}).$$

Since the symmetrical arrangement of MTL and lumped elements is satisfied, the non-diagonal (coupling) elements of **R** and **Z** are zero on both sides, which also results in zero values in the non-diagonal elements of **M** and **Γ**.

**Figure 4:**
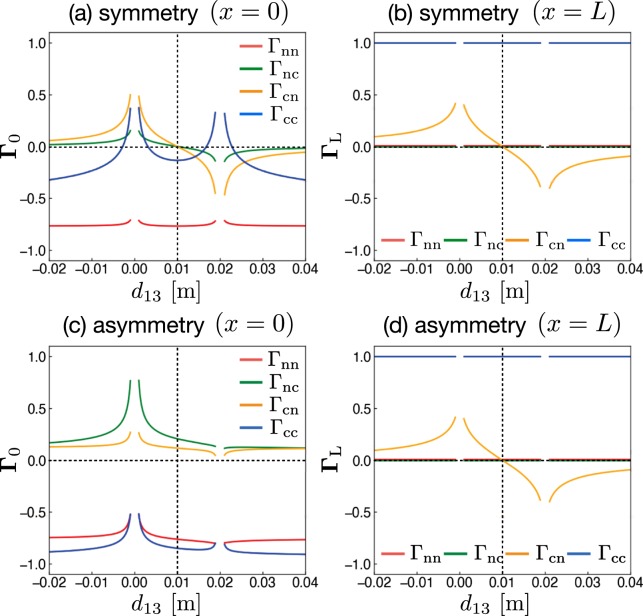
Matrix elements of **Γ**_0_ (*x* = 0) and **Γ**_*L*_ (*x* = *L*) as functions of the distance *d*_13_ between lines 1 − 1′ and 3 − 3′. Two different circuits equivalent to symmetrical (Fig. 3) and asymmetrical (Fig. 1) circuits are used. The distance between lines 1 − 1′ and 2 − 2′ is *d*_12_ = 0.02 m in each circuit. The length and radius of the three lines are *L* = 2 m and 0.5 mm, respectively. The values of the resistances are *R*_12_ = 50 Ω, *R*_23_ = 5 Ω, *R*_13_ = *R*_23_ = 300 Ω, and *R*_12_′ = 349.715 Ω, which is equivalent to the characteristic impedance between lines 1 − 1′ and 2 − 2′(=*Z*_*nn*_).

We explicitly calculate the reflection coefficients **Γ**_0_ and **Γ**_*L*_ using the formula developed herein for various circuit configurations. The results are shown in Fig. 4, where the matrix elements of **Γ**_0_ (*x* = 0) and **Γ**_L_ (*x* = *L*) are shown by changing the distance *d*_13_ between lines 1−1′ and 3−3′. As shown in Fig. 4(a,b) for the symmetric circuit of Fig. 3, the coupling elements of **Γ**_0_ and **Γ**_*L*_ simultaneously become zero at *d*_13_ = 0.01 m, which is the central position of lines 1−1′ and 2−2′. This means that the CM noise is not generated for this case on both sides because the coupling elements are simultaneously zero. On the other hand, as shown in Fig. 4(c,d) for the asymmetric circuit of Fig. 1, the coupling elements of **Γ**_0_ never become zero. This means that CM noise is always generated on the source side because the coupling terms of resistance matrix **R**_0_ always have finite values due to the asymmetric lumped-circuit connection with respect to the ground line of line 3−3′ on the source side, as shown in Fig. 1. Here, Γ_*L*_ has the same behavior as the circuit in Fig. 3 because both circuits have the same condition on the load side. Only Γ_cn_ is dependent on *d*_13_ and becomes zero at *d*_13_ = 0.01 m. On the other hand, Γ_nc_ is always zero because *R*_cc_ becomes infinite on the load side, because line 3–3′ is not connected to other lines. This means that only NM is converted to CM, and not vice versa, on the load side. The above results confirm that the symmetrical configuration in both lumped- and distributed-parameter circuits is the only way to eliminate CM generation.

## Summary

We studied the mechanism of electromagnetic noise generation in symmetric and asymmetric three-line circuits. Telegraph equations, wave equations, and reflection coefficients were derived in the framework of the normal and common modes. The formulation with normal and common mode provides interpretation of the CM noise. We showed that there are two origins of the generation of the electromagnetic noise: (1) coupling during signal propagation in the MTL and (2) coupling at the boundaries of the MTL connected to the lumped-parameter elements.

Although we can discuss CM noise generation in one-dimension (multi-conductor transmission line) problems analytically, this becomes impossible for two or three dimensions. In this case, we are not able to mathematically define the common mode, because the two- or three-dimensional conductors introduce wave propagations in two- and three-dimensional directions, respectively. Hence, we are developing numerical methods by extending the MTL formulation^23^ to two- or three-dimensional circuits for noise analysis of a plane circuit^27^.
